# Elevated levels of faecal calprotectin in primary Sjögren’s syndrome is common and associated with concomitant organic gastrointestinal disease

**DOI:** 10.1186/s13075-015-0907-8

**Published:** 2016-01-12

**Authors:** Kristofer Andréasson, Bodil Ohlsson, Thomas Mandl

**Affiliations:** Section of Rheumatology, Department of Clinical Sciences Lund, Lund University, Lund, Sweden; Section of Internal Medicine, Department of Clinical Sciences Malmö, Lund University, Malmö, Sweden; Section of Rheumatology, Department of Clinical Sciences Malmö, Lund University, Malmö, Sweden

**Keywords:** Faecal calprotectin, Primary Sjögren’s syndrome, Irritable bowel syndrome, Biomarker, Rome III, ESSDAI, ESSPRI

## Abstract

**Background:**

Primary Sjögren’s syndrome (pSS) is a systemic rheumatic disease in which gastrointestinal (GI) symptoms are common. Faecal calprotectin (FC) is a non-invasive biomarker that has been suggested to discriminate organic intestinal disease from functional disorders. The purpose of this study was to explore the usefulness of FC testing in patients with pSS.

**Methods:**

In total, 56 consecutive patients with pSS and 29 healthy control subjects were included in this cross-sectional study. FC was measured with a commercially available enzyme-linked immunosorbent assay kit. GI symptoms were evaluated with the Rome III questionnaire and the Visual Analogue Scale for Irritable Bowel Syndrome. In patients with pSS, disease activity was estimated using the European League Against Rheumatism (EULAR) Sjögren’s Syndrome Disease Activity Index (ESSDAI), and patient-reported outcomes were evaluated with the EULAR Sjögren’s Syndrome Patient-Reported Index.

**Results:**

Patients with pSS had higher levels of FC than healthy control subjects (median 54 μg/g, interquartile range [IQR 20–128]; vs. 20 μg/g [20–43]; *p* = 0.002). Concomitant organic GI disease was found in 14 patients with pSS and included inflammatory bowel disease (*n* = 3), colonic adenoma (*n* = 2) and GI lymphoma (*n* = 1). Patients with organic GI disease had higher FC levels than the other patients with pSS (median 274 μg/g [IQR 61–363] vs. median 34 μg/g [IQR 20–76]; *p* < 0.001). Although patients with pSS reported abdominal discomfort more frequently than healthy control subjects did, such symptoms were not associated with organic GI disease or elevated FC levels. FC correlated moderately with ESSDAI. Excluding patients with organic GI disease, we did not identify any significant association between ESSDAI and FC levels.

**Conclusions:**

GI symptoms are frequent in pSS. Contrary to patient-reported outcomes, elevated FC levels in pSS indicate possible organic GI disease that warrants further investigation.

**Electronic supplementary material:**

The online version of this article (doi:10.1186/s13075-015-0907-8) contains supplementary material, which is available to authorized users.

## Background

Primary Sjögren’s syndrome (pSS) is a systemic autoimmune disease characterized by xerostomia and keratoconjunctivitis sicca with lymphocytic infiltration of the exocrine glands. Typically, the disease affects ocular and oral glands, which is reflected in the current criteria for this disease [1], but pSS can affect several other organs, including the lungs, joints and nervous system, as evaluated using the European League Against Rheumatism (EULAR) Sjögren’s Syndrome Disease Activity Index (ESSDAI) [2]. Gastrointestinal (GI) symptoms have been reported to affect a majority of patients with pSS and are frequently debilitating [3, 4]. Of note, the associations between patient-reported GI symptoms and objective markers of GI dysfunction have been weak [5, 6]. Thus, the majority of patients with pSS have been suggested to have functional GI disorders such as irritable bowel syndrome (IBS) [7–9]. pSS has also been associated with a broad spectrum of organic GI diseases, including reflux esophagitis, chronic atrophic gastritis and coeliac disease, while an association with inflammatory bowel disease (IBD) has been debated [10–13]. Furthermore, patients with pSS are at an overall increased risk of malignancy [14]. Consequently, GI assessment in pSS is challenging. Not least is that it is hard to decide which patients to refer for endoscopy and when.

In primary health care, measurement of faecal calprotectin (FC) has emerged as an objective, easy-to-use biomarker of GI inflammation. Calprotectin is an inflammatory protein also known as S100A8/A9 that is surprisingly inert to degradation and can be readily measured in faeces, even after storage at room temperature for several days [15]. Faecal levels of calprotectin correlate with intestinal migration of neutrophils into the intestinal lumen [16]. Increased levels are seen in GI diseases with an inflammatory component, such as IBD, intestinal malignant and pre-malignant states, and diverticulitis, as well as in upper GI disease [17, 18]. FC is not elevated in patients with functional disorders [17]. FC testing has been validated as a sensitive biomarker for predicting pathological endoscopy in both the upper and lower GI tracts [18, 19]. In a primary health care setting, normal FC testing is increasingly being used to rule out IBD [17, 20]. However, the possible role of FC as a biomarker in patients with pSS is yet to be explored.

The objective of this study was to investigate the usefulness of FC testing in an unselected cohort of patients with pSS.

## Methods

### Subjects

This cross-sectional study was conducted at the Rheumatology Clinic, Skåne University Hospital, Sweden. Consecutive patients with pSS diagnosed according to the American-European Consensus Group criteria for Sjögren’s syndrome were asked to participate [1]. All patients except one agreed to participate in this study and to deliver a stool sample. A control group consisting of hospital workers without any rheumatological or inflammatory GI disease was also included. Characteristics of the 56 patients and 29 control subjects are described in Table 1.

**Table 1:** Patient characteristics

	Patients (*N* = 56)	Control subjects (*N* = 29)
Age, yr	62 (53–68)	56 (49–58)
Males/females	2/54	3/26
Disease duration, yr	5 (7–24)	
Current/prior/never smokers	7/21/28	2/27
Fulfilled AECG criteria for pSS	100 %	
Anti-SS-A–positive	71 %	
Anti-SS-B–positive	39 %	
ANA-positive	73 %	
RF-positive	50 %	
Lip biopsy focus score ≥1	89 %	
ESSDAI	7 (2–7)	
ESSPRI	6 (5–8)	
ESR, mm/h (*n* = 53)	17 (11–28)	
IgG, g/L	13 (10–16)	
C3, g/L	1.0 (0.9–1.2)	
C4, g/L	0.18 (0.13–0.21)	

*AECG* American-European Consensus Group, *ANA* anti-nuclear antibodies, *anti-SS-A* anti-Sjögren’s syndrome–related antigen A, *anti-SS-B* anti-Sjögren’s syndrome–related antigen B; *C3* complement component 3, *C4* complement component 4, *ESR* erythrocyte sedimentation rate, *ESSDAI* European League Against Rheumatism Sjögren’s Syndrome Disease Activity Index, *ESSPRI* European League Against Rheumatism Sjögren’s Syndrome Patient-Reported Index, *IgG* immunoglobulin G, *pSS* primary Sjögren’s syndrome, *RF* rheumatoid factor

Values are given as median (interquartile range), number or percentage

### Clinical assessments

The following data were recorded for all subjects: sex and age at the time of stool delivery. For all patients with pSS, the following data were also retrieved: disease activity according to the ESSDAI [2]; smoking habits; and current medications, including non-steroidal anti-inflammatory drugs (NSAIDs) and proton pump inhibitors (PPIs). Furthermore, the patients’ medical records were scrutinized for concomitant diseases, and any objectively verified chronic organic GI disease or organic GI diagnosis based on endoscopic or radiological examination within 1 year of the time of stool delivery was noted. Serological testing for coeliac disease during the previous 8 years was noted.

### Laboratory analyses

FC was measured in stool samples with a commercially available enzyme-linked immunosorbent assay (ELISA) using a monoclonal antibody (Bühlmann Laboratories, Schönenbuch, Switzerland) [21]. The lower detection limit was 30 μg/g. Analyses were done at the Department of Immunology, Skåne University Hospital, Lund, Sweden. According to the manufacturer of the ELISA and current clinical recommendations, a cut-off value of 50 μg/g was used to discern normal from pathological FC levels. A second cut-off value of 150 μg/g was used to indicate substantial FC elevation in accordance with the latest recommendations [17]. All patients with pSS were further analysed regarding C-reactive protein (CRP), erythrocyte sedimentation rate (ESR), leukocyte count, antibody status and the required assessments for ESSDAI. All laboratory assessments were done blinded to the patients’ medical history.

### Questionnaires

All subjects were asked to fill in the Rome III questionnaire, which is a validated method of identifying functional GI symptoms indicative of IBS [22]. IBS symptom severity was also assessed using the Visual Analogue Scale for Irritable Bowel Syndrome (VAS-IBS). The VAS-IBS reflects abdominal pain, diarrhoea, constipation, bloating and flatulence, vomiting and nausea, psychological well-being and the intestinal symptoms’ influence on daily life as evaluated using seven different VASs, labelled 0–100 mm, where 100 represents absence of symptoms [23]. Finally, sicca, fatigue and pain in patients with pSS were evaluated using the EULAR Sjögren’s Syndrome Patient-Reported Index (ESSPRI) [24].

### Statistics

Median and interquartile range (IQR) were used for descriptive data. For comparison of FC levels between groups, the Mann–Whitney *U* test was used. Fisher’s exact test was used for contingency tables. Spearman’s correlation coefficient was used for correlation analyses. FC values <30 μg/g were approximated to 20 μg/g in all analyses. Because of the interdependence between different questionnaire-based variables analysed in this study, we chose not to use the Bonferroni correction. A two-sided *p* value of 0.05 was considered significant. All analyses were carried out using IBM SPSS Statistics version 20 software (IBM, Armonk, NY, USA).

### Ethical approval

This study was approved by the Regional Ethics Committee, Lund, Sweden (reference number 2011/596). All subjects gave written informed consent according to the Declaration of Helsinki.

## Results

### Increased frequency of pathological FC testing in pSS

**Fig. 1:**
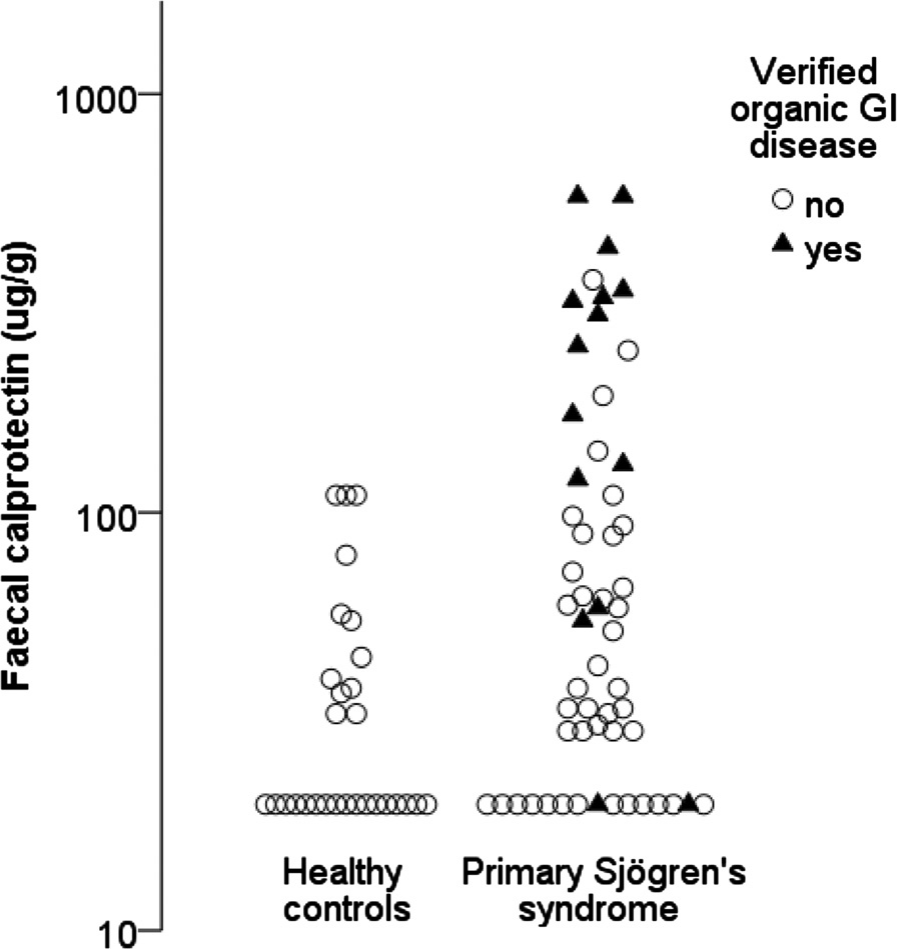
Elevated levels of faecal calprotectin in primary Sjögren’s syndrome. Faecal calprotectin levels were higher in patients with primary Sjögren’s syndrome (*n* = 56) than in healthy control subjects (*n* = 29) (*p* = 0.002 by Mann–Whitney *U* test). Subjects with a concomitant organic gastrointestinal (GI) disease are indicated by *closed triangles*; other subjects are represented by *open circles*

Patients with pSS had significantly higher levels of FC than healthy control subjects (median 54 μg/g [IQR 20–128] vs. 20 μg/g [20–43], *p* = 0.002) (Fig. 1). Among the patients with pSS, 52 % (29 of 56) had pathological FC levels (>50 μg/g) and 21 % (12 of 56) had substantial FC elevation (>150 μg/g). Corresponding numbers among healthy control subjects were 20 % and 0 %, respectively.

### Organic gastrointestinal disease is common in patients with pSS and pathological FC levels

Of the 56 patients with pSS, 14 had a concomitant organic GI disease, including 1 patient with extensive lymphoma affecting the GI tract (Table 2). Patients with organic GI disease had significantly higher FC levels than the other patients (274 μg/g [61–363] vs. 34 μg/g [20–76], *p* < 0.001), and 75 % of patients (9 of 12) with FC >150 μg/g had organic GI disease. The corresponding percentage for patients with FC levels between 50 and 150 μg/g was 18 % (3 of 17), and for patients with FC within normal range it was 7 % (2 of 27) (Fig. 1). Excluding patients with organic GI disease, patients with pSS still had slightly higher levels of FC than healthy control subjects (34 μg/g [20–76] vs. 20 μg/g [20–43], *p* = 0.036). Of note, one patient not categorized as having organic GI disease but with elevated FC (120 μg/g) was diagnosed with mucosa-associated lymphoid tissue lymphoma in the ventricle 18 months after FC testing.

**Table 2:** Concomitant organic GI diseases in patients with pSS

Diagnosis	Number of affected patients
Inflammatory bowel disease, *of which*	3
Ulcerative colitis	*1*
Microscopic colitis	*1*
Unspecified rectal inflammation	*1*
Chronic atrophic gastritis	3
Colonic adenoma	2
Gastrointestinal lymphoma	1
Duodenitis	1
Coeliac disease	2^a^
Gastric antral vascular ectasia	1
Diverticulitis	1^a^
Gastric binding with surgical complication	1
Total	14

^a^One patient had both coeliac disease and diverticulitis

### FC and other clinical markers of disease

Patients with organic GI disease had higher ESSDAI scores than other patients (10 [4–12] vs. 6 [1–9], *p* = 0.121). In the whole group, FC correlated with ESSDAI (*r*_s_ = 0.32, *p* = 0.015). When patients with organic GI disease were excluded, the correlation between FC and ESSDAI became non-significant (*r*_s_ = 0.28, *p* = 0.074).

Further analyses failed to show any difference in FC levels between users and non-users of NSAIDs (38 vs. 62 μg/g, *p* = 0.168) and PPIs (63 vs. 34 μg/g, *p* = 0.135). In contrast, FC correlated with biochemical markers of systemic disease, such as CRP (*r*_s_ = 0.37, *p* = 0.006) and ESR (*r*_s_ = 0.34, *p* = 0.012), but not with immunoglobulin G or complement components 3 and 4 (*r*_s_ = −0.12, *p* = 0.363; *r*_s_ = 0.24, *p* = 0.070; and *r*_s_ = −0.07, *p* = 0.606, respectively).

### FC elevation is independent of patient-reported bowel discomfort in pSS

GI symptoms were evaluated with the VAS-IBS and the Rome III questionnaire, and questionnaire data were available for 21 control subjects and 53 patients with pSS. Bowel discomfort of all kinds was common in pSS (Tables 3 and 4). Of note, 37 of 51 patients without IBD had a symptomatology indicative of a functional GI disorder. Results from both questionnaires indicated a substantial heterogeneity encompassing a plethora of GI symptoms in the pSS group. We did not see any relationship between FC levels and patient-reported GI symptoms (Tables 3 and 4). Furthermore, we were unable to identify any association between questionnaire data and organic GI disease (see Additional file 1). In contrast, average results from the VAS-IBS correlated with ESSPRI (*r*_s_ = −0.56, *p* < 0.001). Patients with IBS had more pain, as evaluated by the ESSPRI-Pain sub-scale score, than patients with pSS without IBS (8 vs. 5, *p* = 0.013). Four patients in the study had a clinical diagnosis of fibromyalgia. All of these patients had a concomitant functional GI disorder.

**Table 3:** Gastrointestinal discomfort is common in primary Sjögren’s syndrome, based on Visual Analogue Scale for Irritable Bowel Syndrome results

Domain	Control subjects (*N* = 21)	pSS with FC <50 μg/g (*N* = 25)	pSS with FC >50 μg/g (*N* = 25)
Abdominal pain	97	87^a^	88^b^
Diarrhoea	100	94^b^	91^b^
Constipation	96	78^a^	80^a^
Bloating and flatulence	90	77^a^	61^b^
Vomiting and nausea	100	97^b^	97^b^
Psychological well-being	93	72^b^	89
Influence on daily life	99	94^a^	68^b^
Average	93	82^a^	78^b^

*FC* faecal calprotectin, *pSS* primary Sjögren’s syndrome

Values are given as medians in millimetres, and higher values indicate less symptoms. Mann–Whitney *U* test was used to compare groups. Significant differences were identified between control subjects in comparison with both pSS groups. No significant differences were identified between patients with pSS with and without pathological FC testing

^a^ *p* < 0.05 compared with control subjects

^b^ *p* < 0.01 compared with control subjects

**Table 4:** Gastrointestinal discomfort is common in primary Sjögren’s syndrome according to Rome III criteria

Symptomatology	Control subjects (*N* = 23)	Patients with FC <50 μg/g (*n* = 26)	Patients with FC >50 μg/g (*n* = 25)	All patients with pSS (*N* = 51)
Functional heartburn	1/23	7/26	6/25	13/51
Functional dysphagia	1/23	4/26	4/25	8/51
Functional dyspepsia	1/23	10/26^a^	9/25^b^	19/51^a^
Irritable bowel syndrome	3/23	8/26	8/25	16/51
Functional constipation	3/23	2/26	0/25	2/51
Faecal Incontinence	1/23	2/26	8/25^b^	10/51
Any of above	8/23	17/26^b^	20/25^a^	37/51^a^

*FC* faecal calprotectin; pSS primary Sjögren’s syndrome

Data shown are number of subjects with data indicative of functional gastrointestinal disorders. Significant differences were identified between control subjects in comparison to both primary Sjögren’s syndrome groups. Fisher’s exact test was used to compare groups. No significant differences were identified between patients with pSS with and without pathological FC testing. According to the Rome III criteria, organic disease must be ruled out in order to receive a diagnosis of functional gastrointestinal disorder

^a^ *p* < 0.05 compared with control subjects

^b^ *p* < 0.01 compared with control subjects

## Discussion

GI symptoms are common in pSS but not always associated with organic GI disease [3, 6]. In this study, we found higher levels of FC in patients with pSS than in healthy control subjects, especially among patients with organic GI co-morbidities such as IBD, colonic adenomas and malignancies. To our knowledge, this is the first extended report on FC in patients with pSS [25].

FC is a validated biomarker of inflammation. In the field of gastroenterology, FC is now a routine analysis in the management of IBD in Europe. An ELISA-based method for FC analysis has recently received U.S. Food and Drug Administration approval, and FC is now readily available in the clinic also in North America [17, 20]. FC is a feasible biomarker, which might explain the high participation rate in this study of consecutive patients [17].

Today, FC is also frequently used in primary health care, especially in relation to patients being referred for endoscopy [19]. Elevated levels of FC are associated not only with IBD but also with malignant and pre-malignant GI diseases as well as upper GI pathologies such as reflux oesophagitis and ulcers and/or erosions. Of note, all conditions in Table 2 have previously been reported to be associated with pathological FC testing [17, 19, 26, 27].

The origin of FC in pSS is unclear. In this study, we found elevated FC levels in patients with pSS both with and without additional organic GI disease compared with levels in healthy control subjects. We suggest that FC levels >150 μg/g cannot routinely be explained by pSS alone, as 9 of 12 patients with FC >150 μg/g had a concomitant organic GI disease. The situation may be different for FC levels between 50 and 150 μg/g, for which organic GI pathology was identified in only 3 of 17 patients. We can only speculate as to what degree GI involvement of pSS contributes to the increased FC levels reported in patients without known organic disease. Use of both PPIs and NSAIDs has been suggested to cause a slight increase in FC [28], but we did not find any such associations that might explain our results.

Previous studies have shown increased levels of calprotectin in saliva from patients with pSS, suggesting that calprotectin may originate from the exocrine glands or may be released in the oral cavity from dry and inflamed mucosal tissues, possibly both in the GI tract and in the airways [29]. By analogy to saliva, calprotectin in faeces could originate from exocrine glands in the bowel. The tendency toward correlations between ESSDAI and FC as well as between ESR and FC suggest that FC could be a marker of disease activity and could possibly be used in pSS to follow treatment effects in a similar way as in IBD. Furthermore, the average results from the VAS-IBS correlated with patient-reported disease activity measured with the ESSPRI. The aetiology of functional GI disorders is unknown, but the diseases are characterized by absence of objective, pathological findings [28]. It remains to be determined whether GI symptoms in pSS should be defined as functional GI disorders or as a separate entity of GI disorder associated with pSS.

Some methodological issues have been raised regarding the measurement of FC, not least because different antibodies are used in the different ELISAs available [21, 30]. The ELISA used in this study has been thoroughly reviewed and suggested to be superior to some other ELISAs, although conflicting data have been presented [21, 30, 31].

This study has important limitations. Not all patients had been subject to invasive GI examination, and only procedures carried out within 1 year of the FC measurement were considered valid for this study, together with a previous diagnosis of chronic GI disease. It is possible, therefore, that we underestimated the true prevalence of organic GI disease in the cohort. In addition, this study comprised only 56 patients with pSS. Further prospective studies involving larger patient cohorts that include routine endoscopy are needed before any firm conclusions can be drawn regarding the pathophysiology behind the elevated FC in pSS.

The prevalence of coeliac disease has been estimated to be 5 % among patients with pSS [32]. It should be noted that previous studies have suggested that serological testing is superior to FC in identifying coeliac disease in adults [33].

In the field of rheumatology, elevated FC levels have been reported in systemic sclerosis and ankylosing spondylitis, while knowledge on FC levels in RA is limited [25, 34, 35]. The role of FC testing in this field is not yet established, and further studies including FC data on patients with various rheumatic diseases are warranted.

## Conclusions

GI symptoms in pSS are common. Regardless of symptoms, FC elevation, especially if >150 μg/g, may indicate concomitant organic GI disease (e.g., malignancy or IBD), which warrants further investigation.

## Additional file

Additional file 1:
**Table S1a and S1b.** These tables present GI symptoms in patients with pSS with and without concomitant organic GI disease. (DOCX 15 kb)

## Abbreviations

AECG: American-European Consensus Group
ANA: anti-nuclear antibodies
anti-SS-A: anti-Sjögren’s-syndrome-related antigen A
anti-SS-B: anti-Sjögren’s-syndrome-related antigen B
C3: complement component 3
C4: complement component 4
CRP: C-reactive protein
ELISA: enzyme-linked immunosorbent assay
ESR: erythrocyte sedimentation rate
ESSDAI: European League Against Rheumatism Sjögren’s Syndrome Disease Activity Index
ESSPRI: European League Against Rheumatism Sjögren’s Syndrome Patient-Reported Index
EULAR: European League Against Rheumatism
FC: faecal calprotectin
GI: gastrointestinal
IBD: inflammatory bowel disease
IBS: irritable bowel syndrome
IgG: immunoglobulin G
IQR: interquartile range
NSAID: non-steroidal anti-inflammatory drug
PPI: proton pump inhibitor
pSS: primary Sjögren’s syndrome
RF: rheumatoid factor
VAS-IBS: Visual Analogue Scale for Irritable Bowel Syndrome
