# Supplementary material for: Elevated levels of faecal calprotectin in primary Sjögren’s syndrome is common and associated with concomitant organic gastrointestinal disease
Source: Arthritis Res Ther. 2016 Jan 12;18:9. doi: 10.1186/s13075-015-0907-8 (PMC4718038; doi:10.1186/s13075-015-0907-8)
Supplement: Additional file 1: — Table S1a and S1b. These tables present GI symptoms in patients with pSS with and without concomitant organic GI disease. (DOCX 15 kb) [file 13075_2015_907_MOESM1_ESM.docx]

Supplemental table 1A. Median Visual Analogue Scale for Irritable Bowel Syndrome values in primary Sjögren’s syndrome patients with and without organic gastrointestinal (GI) disease. No significant differences were identified between these patient groups. P>0.1 for all comparisons.

| **Domain** |  | Patients without organic GI disease n=38 |  | Patients with organic GI disease n=12 |
| --- | --- | --- | --- | --- |
| Abdominal pain |  | 86 |  | 95 |
| Diarrhoea |  | 92 |  | 91 |
| Constipation |  | 77 |  | 91 |
| Bloating and flatulence |  | 72 |  | 54 |
| Vomiting and nausea |  | 97 |  | 98 |
| Psychological well-being |  | 73 |  | 88 |
| Influence on daily life |  | 89 |  | 84 |
| **Average** |  | **81** |  | **87** |

GI: gastrointestinal

Supplemental table 1B. Number of subjects with symptoms indicative of functional gastrointestinal (GI) disorders according to the ROME III criteria§. No significant differences were identified between pSS patients with and without organic GI disease. P>0.1 for all comparisons.

| **Symptomatology** |  | Patients without organic GI disease n=41 |  | Patients with organic GI disease n=12 |
| --- | --- | --- | --- | --- |
| Functional Heartburn |  | 10/41 |  | 4/12 |
| Functional Dysphagia |  | 6/41 |  | 2/12 |
| Functional Dyspepsia |  | 16/41 |  | 4/12 |
| Irritable Bowel Syndrome |  | 14/41 |  | 4/12 |
| Functional constipation |  | 2/41 |  | 0/12 |
| Faecal Incontinence |  | 8/41 |  | 3/12 |
| **Any of above** |  | **31/41** |  | **8/12** |

§ *According to the ROME III criteria, organic disease must be ruled out in order to receive a diagnosis of functional gastrointestinal disorder.*GI: Gastrointestinal
